# Diet-related chronic disease in the northeastern United States: a model-based clustering approach

**DOI:** 10.1186/s12942-015-0017-5

**Published:** 2015-09-04

**Authors:** Abby Flynt, Madeleine I. G. Daepp

**Affiliations:** Department of Mathematics, Bucknell University, 701 Moore Ave, 17837 Lewisburg, PA USA; Integrated Studies in Land and Food Systems, The University of British Columbia Vancouver, 2329 West Mall, V6T 1Z4 Vancouver, BC Canada

**Keywords:** Obesity, Diabetes, Food access, Socioeconomic status, Model-based clustering, Variable selection

## Abstract

**Background:**

Obesity and diabetes are global public health concerns. Studies indicate a relationship between socioeconomic, demographic and environmental variables and the spatial patterns of diet-related chronic disease. In this paper, we propose a methodology using model-based clustering and variable selection to predict rates of obesity and diabetes. We test this method through an application in the northeastern United States.

**Methods:**

We use model-based clustering, an unsupervised learning approach, to find latent clusters of similar US counties based on a set of socioeconomic, demographic, and environmental variables chosen through the process of variable selection. We then use Analysis of Variance and Post-hoc Tukey comparisons to examine differences in rates of obesity and diabetes for the clusters from the resulting clustering solution.

**Results:**

We find access to supermarkets, median household income, population density and socioeconomic status to be important in clustering the counties of two northeastern states. The results of the cluster analysis can be used to identify two sets of counties with significantly lower rates of diet-related chronic disease than those observed in the other identified clusters. These relatively healthy clusters are distinguished by the large central and large fringe metropolitan areas contained in their component counties. However, the relationship of socio-demographic factors and diet-related chronic disease is more complicated than previous research would suggest. Additionally, we find evidence of low food access in two clusters of counties adjacent to large central and fringe metropolitan areas. While food access has previously been seen as a problem of inner-city or remote rural areas, this study offers preliminary evidence of declining food access in suburban areas.

**Conclusions:**

Model-based clustering with variable selection offers a new approach to the analysis of socioeconomic, demographic, and environmental data for diet-related chronic disease prediction. In a test application to two northeastern states, this method allows us to identify two sets of metropolitan counties with significantly lower diet-related chronic disease rates than those observed in most rural and suburban areas. Our method could be applied to larger geographic areas or other countries with comparable data sets, offering a promising method for researchers interested in the global increase in diet-related chronic disease.

**Electronic supplementary material:**

The online version of this article (doi:10.1186/s12942-015-0017-5) contains supplementary material, which is available to authorized users.

## Background

The world has seen a dramatic increase in the prevalence of diet-related chronic disease in recent decades [1, 2]. Diet-related chronic diseases—preventable illnesses for which poor diet quality is an important risk factor [3]—include obesity and diabetes, diseases estimated to affect over 13 and 9 % of adults worldwide, respectively [4]. These diseases pose significant public health concerns: adults diagnosed with diabetes have 1.5 times the death rate of adults who have not been diagnosed with the disease [5], and obesity is associated with numerous comorbidities including hypertension, coronary heart disease, and a generally increased risk of all-cause mortality [6, 7].

In the United States, where one in three adults now qualify as obese [8] and nearly one in ten suffer from diabetes [5], researchers have identified geographic patterns in the prevalence of diet-related chronic disease. Jackson et al. [9] found that residents of rural counties were significantly more likely to report being overweight or obese. Similarly, diabetes rates are particularly high in rural Appalachian and southern counties [10]. Recent analysis has suggested that the patterns may be more complex: while researchers continued to observe particularly high obesity rates in rural southern counties, lower obesity rates were seen in metropolitan and non-metropolitan counties elsewhere in the United States [11].

Researchers have identified a number of population-level risk factors for obesity and diabetes, but these factors have largely heterogenous spatial distributions and thus cannot easily explain differences in disease prevalence between rural and urban counties. Many public health experts consider the food environment–the grocery stores, restaurants and other food vendors comprised in the built environment—a likely contributor to expanding American waistlines [12, 13]. People with easy access to supermarkets are more likely to consume fruits and vegetables and less likely to be obese than comparable people with lower access [12, 14, 15]. The distribution of supermarkets and grocery stores across the United States, examined in a report issued by the United States Department of Agriculture’s Economic Research Service [16], varies significantly across US census tracts. Although the report did identify low-income census tracts with a dearth of supermarkets (i.e. “food deserts” [17]) in rural areas, residents of very dense census tracts with high poverty levels were also likely to have limited access to supermarkets or grocery stores.

Multiple studies show a significantly higher prevalence of diet-related chronic disease among minority groups [9, 18, 19]. Obesity and type 2 diabetes have been found to be strongly related with measures of socioeconomic status, with the highest disease rates occurring in groups with the lowest levels of education or income [18]. These socioeconomic and demographic variables associated with rates of diet-related disease may compound the effects of place [20].

In this paper, we use statistical clustering analysis as a means of deconstructing the roles of the aforementioned socioeconomic, demographic, and environmental risk factors as contributors to observed patterns of obesity and diabetes prevalence in US counties. Although the clustering of risk factors to obtain meaningful classifications of spatial geographies has been the subject of extensive study in geography [21–23], open questions include which attributes of a population are integral to the identification of different subpopulations and how to determine whether a geographic classification describes a meaningful difference in classified groups [24, 25]. We apply the method of model-based clustering with variable selection as an empirical approach to the identification of relevant population-level risk factors and the classification of clusters related to the distributions of these risk factors.

Model-based clustering is a popular clustering method that has been used in a variety of application areas outside of geography. Some examples include gene expression modeling [26], food authenticity studies [27], social network modeling [28] and identification of galaxy properties [29]. The method is based on a probability model unlike other common clustering algorithms, which are more heuristically motivated. In the latter algorithms, practitioners need to make important decisions regarding the types of models to fit as well as determine the “best” number of clusters for the data. Both of these choices greatly impact the final clustering solution and thus any conclusions made from the final model. In model-based clustering however, the choice of the “best” clustering solution is just a model choice problem that can be solved by using readily available statistical methods.

The aim of this study is to present model-based clustering with variable selection as a means of identifying and classifying risk factors relevant to population health patterns. We apply this method to assess whether there is a meaningful distinction in the socioeconomic, demographic, and environmental characteristics of US counties associated with residents’ susceptibility to obesity and diabetes as a case study of this empirical approach. We test the method’s efficacy by examining the value of the resulting clusters for predicting the spatial distributions in the rates of diet-related chronic disease.

## Methods

### Data

#### Study region

This study uses data from the US states of Pennsylvania (PA) and New York (NY) to test the predictive value of the clustering methodology. PA and NY are two northeastern states with approximately 12.8 million and 19.7 million inhabitants, respectively [30]. The states both have high obesity rates (state-level obesity topped 30 % in PA in 2013, while NY had a rate of 25.4 % [31]) and significant within-state variance in these rates. While two-sample t-tests conducted with the obesity, diabetes, and median household income variables confirm that each variable has significantly different means in each state, the means of other socioeconomic, demographic, and environmental variables were not significantly different across states.

#### Socioeconomic and demographic variables

**Fig. 1:**
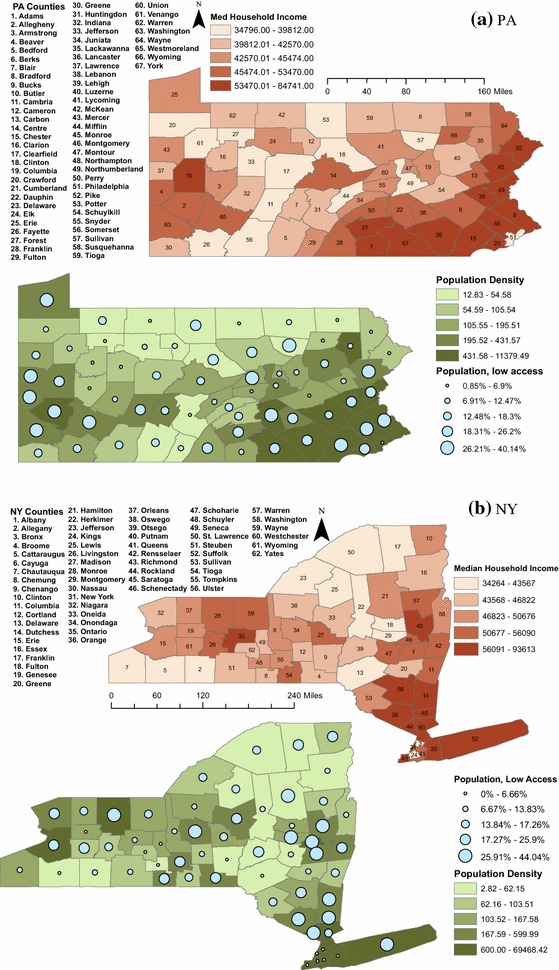
County-level median household income, population density, and grocery store access, quintiles. PA (**a**) and NY (**b**) both comprise counties with varying levels of each of our clustering variables. The counties in New York City have the highest population densities of our data set, but the range in the percentage of residents with low access to grocery stores and supermarkets and in median household income is comparable to that seen across other counties of both states

We examined a number of socioeconomic and demographic variables identified by public health researchers as possible risk factors for obesity and diabetes. Specifically, we looked at unemployment, median household income, and a socioeconomic status (SES) index based on education levels, household composition, race, and poverty rates. County level unemployment rates for 2012 were obtained from the US Department of Labor Bureau of Labor Statistics [32]. Median household income, estimated for 2012, and population density, from the 2010 census, were measured by the US Census Bureau [33]. Summary statistics for these measures can be found in Table 1 and the geographic distributions can be seen in Fig. 1a for PA and Fig. 1b for NY.

**Table 1:** Summary statistics of demographic, socioeconomic, and access variables for PA & NY

Variable	Mean	SD	Min.	1st Qu.	Median	3rd Qu.	Max.
Unemployment (%)	8.68	1.23	6.00	7.80	8.60	9.40	13.70
Pop. density (pop/m^2^)	1691.58	7636.93	2.82	71.17	127.24	373.90	69,468.42
Median HH income ($)	49,367	11,405	34,264	42,363	46,190	52,659	93,613
SES index	0.00	3.17	−5.18	−1.32	−0.44	0.69	22.42
Low access (%)	16.40	10.24	0.00	9.18	14.65	23.67	44.04

Prior research suggests that no single variable can adequately quantify socioeconomic status [34]. To address the multidimensional nature of SES, this study incorporated an SES index modeled on a number of existing indices of socioeconomic deprivation including the Townsend Index, the Jarman Underprivileged Area (UPA) Score, and the Carstairs Index [35–37]. Deprivation indices, described below, have been used by a number of researchers as a means of including multiple determinants of socioeconomic status in statistical analyses [38–42].

Originally developed for use in the United Kingdom, the Townsend Index is among the most commonly used indices of deprivation [35, 39]. It is constructed from the unemployment rate, a measure of “overcrowded” households, the percentage of households without car ownership, and the percentage of renters [43]. The Carstairs Index is similar, replacing the measurement of renters with “proportion low social class”, while the Jarman UPA Score replaces the car ownership and non-homeowner variables with demographic measures including the proportion of single parent and lone pensioner households and recent immigrants.

Many of these indices are not easily translatable to research in the US. Variables such as social class are not measured by the US Census Bureau, while other variables (e.g. crowding) have not been found to correlate with physical health in the US context [39]. These concerns have been addressed by more recent studies: researchers in Canada have constructed indices incorporating the percent low-income households, percent single parent households, percent immigrants and/or measures of low educational attainment in place of the class, homeownership, crowding, or car ownership variables used in the UK indices [40–42]. An additional concern is the strong evidence of ethnic or racial disparities in health in the United States, which suggests that any US index should incorporate a measure of area ethnic or racial makeup [44]. For this study, we construct an index modeled on recent updates to the major British indices that is additionally adjusted to be appropriate for research specific to the US.

**Fig. 2:**
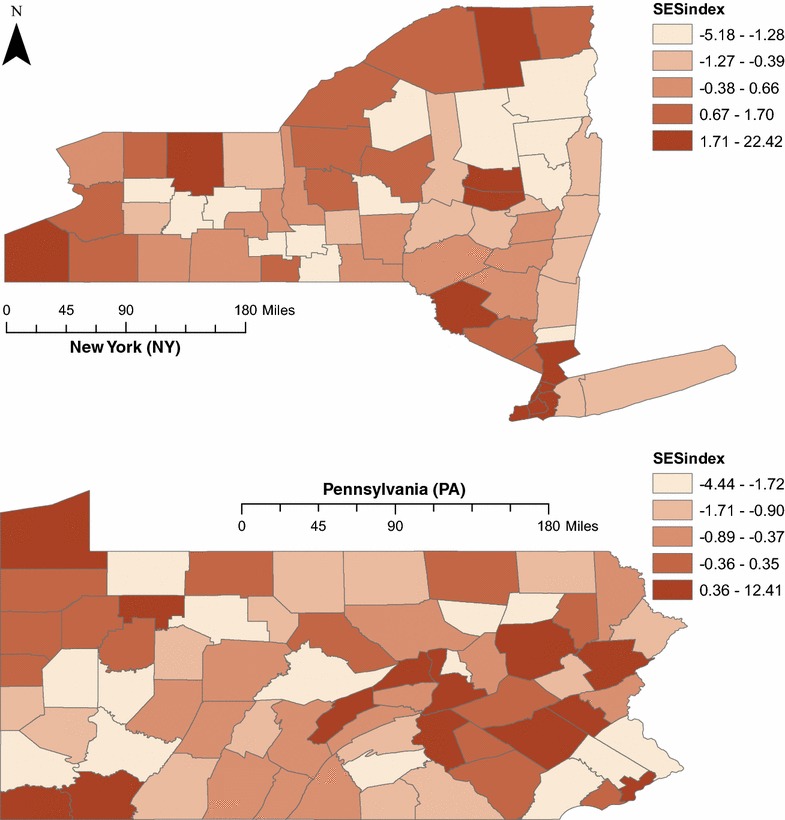
SES Index, Quintiles. Counties in both states vary in level of deprivation, as measured in SES index quintiles

The SES Index is defined as the sum of the standardized scores of four variables: (1) the percent of total county adults over age 25 with less than a high school degree, (2) the percent of households headed by single females, (3) the percent non-white county residents, and (4) the poverty rate. The first three variables were obtained from the 2008–2012 American Community Survey [45]; percent non-white was calculated as 100 minus the percent of the county-population that self-identified as non-Hispanic White. The poverty rate was obtained from the US Census Bureau’s Small Area Income and Poverty Estimates for 2012 [33]. While the variables are correlated (see Table 2) the use of multiple measures, rather than any single measure of socioeconomic status, allows us to identify regions where multiple sources of socioeconomic deprivation interact (see Fig. 2).

**Table 2:** Pearson correlations for variables used in the socioeconomic index

	(1)	(2)	(3)	(4)
1. Education less than high school (%)	1.000			
2. Single female-headed households (%)	0.538	1.000		
3. Non-white county residents (%)	0.476	0.724	1.000	
4. Poverty rate	0.690	0.648	0.493	1.000

#### Environmental variables

Variables related to the built environment are also associated with diet-related chronic disease. In particular, access to healthy foods may affect an individual’s ability to maintain a healthy diet [12, 15, 17] and, in the United States, rural counties have been identified as having higher obesity prevalences than do more urbanized counties [9].

Food access was measured as the percent of each county’s population living in a “food desert,” a neighborhood without supermarkets or large grocery stores in 2010. This variable, obtained from the US Department of Agriculture’s Food Environment Atlas [46], is defined as the percent of individuals in urban census blocks living more than one mile from a major supermarket or grocery store and the percent in rural census blocks living more than 10 miles from such stores, aggregated to the county level. That is, a county with a “high” percent low access is considered to have limited food access. Census blocks are dichotomized into the urban and rural classifications following US Census Bureau’s Urban Area Definition [47]. Supermarkets and large grocery stores were defined as stores with all the major food departments (dairy, bakery, butcher, produce, and delicatessen) of a traditional supermarket that reported at least $2 million in annual sales.

To examine our results by county type, we used classifications from the 2013 National Center for Health Statistics (NCHS) Urban-Rural Classification Scheme [48]. The NCHS classifies counties into six categories, detailed in Table 3, which were created for researchers and practitioners interested in the health differences found across areas with varying levels of urbanization. The two states examined in our study, PA and NY, both have a mix of counties from across the different classifications.

**Table 3:** NCHS urban–rural classification scheme for counties

Urbanization level	Classification rule
Metropolitan counties	
Large central metro	Populations of 1 million or more that
	1. Contain the entire population of the largest principal city, or
	2. Have their entire population contained in the largest principal city, or
	3. Contain at least 250,000 inhabitants of any principal city
Large fringe metro	Populations of 1 million or more that did not qualify as large central metro counties
Medium metro	Populations of 250,000–999, 999
Small metro	Populations of 50,000–250,000
Non-metropolitan counties	
Micropolitan	Populations of 10,000–49,999
Noncore	Populations less than 10,000

#### Health variables

Two health outcomes are examined in this study: (1) obesity prevalence and (2) diabetes prevalence. Obesity prevalence is defined as the percent of adults in each county reporting a body mass index of at least 30 in 2010. The age-adjusted estimates, calculated by the Centers for Disease Control and Prevention (CDC) with data from the Behavioral Risk Factor Surveillance System (BRFSS) for 2008–2010 and the US Census Bureau, were determined through the use of small-area statistical modeling [49, 50]. The adult diabetes rate is an estimate of the age-adjusted percent of the adult county population living with diabetes, also determined with CDC BRFSS and US Census Bureau data with Bayesian small area estimation [51, 52]. Summary statistics for these variables can be found in Table 4.

**Table 4:** Summary statistics of health variables, unstandardized

Variable	Mean	SD	Minimum	1st Quartile	Median	3rd Quartile	Maximum
Percent obesity	28.90	3.24	15.70	27.40	29.00	31.20	36.20
PA only	30.28	2.67	22.20	28.65	30.80	32.15	36.20
NY only	27.40	3.17	15.70	25.95	28.10	29.15	33.90
Percent diabetes	10.13	1.22	7.20	9.30	10.00	11.00	13.00
PA only	10.64	1.24	7.30	9.80	10.70	11.30	13.00
NY only	9.59	0.95	7.20	9.00	9.70	10.10	11.80

### Analysis

To look at the potential distinctions of US counties in rates of obesity and diabetes, we employ a method of statistical clustering known as model-based clustering. This type of analysis will allow us to identify subpopulations of counties particularly susceptible to diet-related chronic disease, based on the previously mentioned socioeconomic, demographic and environmental variables.

#### Cluster analysis

Model-based clustering was first introduced by Wolfe in 1963 [53], and is further discussed by Banfield and Raftery [54], McLahlan and Peel [55] and Fraley and Raftery [56]. The underlying idea of model-based clustering is that the observed data in a population actually come from several subpopulations, which we can model separately. Then using finite mixture models, the overall population is modeled as a mixture of these subpopulations.

A mixture model is a probabilistic weighted combination of subpopulations within an overall population. If we consider K possible subpopulations, let ${\mathbf {y}}$ be the dependent variable from density *f* parametrized by $\theta _k$, and $\pi _k$ be the prior probability distribution for subpopulation *k*. Then the general form for a finite mixture model that has *K* subpopulations is

$$\begin{aligned} f({\mathbf {y}}) = \sum _{k=1}^K \pi _k f({\mathbf {y}}|\theta _k), \end{aligned}$$

where

$$\begin{aligned} \pi _k \ge 0, \quad \sum _{k=1}^K \pi _k = 1. \end{aligned}$$

Often, it is assumed that each subpopulation follows a Gaussian distribution and $f({\mathbf {y}})$ is a mixture of Gaussians. Now, thinking of the subpopulations as clusters, the mixture model can be partitioned into clusters using Bayes’ rule. Bayes’ rule provides estimates for the posterior probability that each observation belongs to cluster $k, \ k= 1, \ldots , K$, namely

$$\begin{aligned} P(k|{\mathbf {y}}) = \frac{\pi _k f({\mathbf {y}}|\theta _k)}{\sum _j\pi _j f({\mathbf {y}}| \theta _j) }. \end{aligned}$$

Thus, observation ${\mathbf {y}}$ is assigned to cluster *k*, if $P(k|{\mathbf {y}})>P(k'|{\mathbf {y}})$, $\forall \ k'\ne k$. To estimate the parameter vector $\mathbf {\theta } = \{\theta _1, \ldots , \theta _K\}$, we maximize the likelihood function using the Expectation-Maximization (EM) algorithm [57]. Assuming there are N observations, the log-likelihood function is given by

1 $$\begin{aligned} \ell (\theta ) = \sum _{n=1}^N \log f(y_n) = \sum _{n=1}^N \log \left( \sum _{k=1}^K \pi _k f(y_n| \theta _k) \right) . \end{aligned}$$

The E-step of the algorithm is to estimate the posterior class probabilities for each observation $\hat{p}_{nk} = P(k|y_n)$ and then derive the prior class probabilities using the estimates, $\hat{\pi }_k = \frac{1}{N}\sum _{n=1}^N\hat{p}_{nk}$. Next, the M-step is to maximize the log-likelihood separately for each component using $\hat{p}_{nk}$ as weights

$$\begin{aligned} \max _{\theta _k} \sum _{n=1}^N \hat{p}_{nk} \log f(y_n|\theta _k). \end{aligned}$$

The algorithm then iterates between steps until the improvement of the log-likelihood function meets the desired tolerance level or the algorithm has reached a maximum number of iterations. As previously stated, each observation is assigned to the cluster that has the maximum posterior probability. Choosing the number of clusters *K* is a statistical model selection problem decided by some type of information criterion. The criterion that is often used for selecting *K*, is the Bayesian Information Criterion (BIC) [58] which takes the form

$$\begin{aligned} BIC = 2 \hat{\ell }(\theta ) - p \log (N), \end{aligned}$$

where $\hat{\ell }(\theta )$ is the maximized log-likelihood from (1) and *p* is the number of parameters estimated. In model fitting, increasing the number of parameters estimated can increase the log-likelihood of the model, thus the BIC penalizes the log-likelihood by the number of estimated parameters. The model that produces the smallest BIC is chosen as the “best” clustering solution. To implement the approach of model-based clustering, the popular package **mclust** is used within the statistical software R [59].

#### Variable selection

While we have chosen several socioeconomic, demographic, and environmental variables to use in our model, it is not necessarily the case that they all contribute to the clustering structure present in the data. These unnecessary variables can make it difficult to fit the model or even degrade the clustering solution. For example, when two variables are correlated and forced into a model together, they can produce a clustering solution with a higher BIC than a model that included only one of the correlated variables. Variable selection helps avoid this problem by recognizing that only one of the correlated variables is useful for clustering the data. Moreover, the inclusion of extra variables in clustering can greatly reduce the interpretability and visualization of the final clustering solution.

There are several procedures available for variable selection with model-based clustering, such as those proposed by Raftery and Dean [60], Maugis et al. [61] and Andrews and McNicholas [62]. All three of these variable selection techniques can be implemented in R using the packages **clustvarsel**, **VarSelLCM** and **vscc** respectively. The latter two procedures did not result in a reduction of our variable space and practically produced less meaningful clusters of data (discussed in “Results”). Thus, before using model-based clustering, we implement the variable selection procedure of Raftery and Dean to determine the important clustering variables. This procedure is a greedy search algorithm that searches for the variable to add to the model that most improves the clustering solution as measured by the BIC. It then determines whether one of the current clustering variables can be dropped from the model, and stops when there is no improvement in the clustering solution. The algorithm is summarized as follows:

1. Select the first clustering variable that provides the most evidence of clustering.
2. Select the second clustering variable that shows the most evidence of clustering including the first selected variable.
3. Propose the next clustering variable that shows the most evidence of clustering including the first two selected variables. Include this variable only if there is an improvement in the clustering solution.
4. Propose a variable for removal from the set of clustering variables that produces the weakest evidence for inclusion in the clustering. Remove this variable from the set of clustering variables if the evidence for clustering without it is stronger than that of clustering with it.
5. Iterate between steps 3 and 4 until two consecutive steps have been rejected. Stop once this occurs.

As stated in [62], one of the main problems with the **clustvarsel** package of Raftery and Dean is that it can be very slow in high-dimensions. As we were dealing with 5 variables and 129 counties, we did not find computation time to be an issue. Using the variables chosen by this process, we then determine the final clustering solution using model-based clustering as described above; results were mapped to county boundaries (US Census Bureau 2010 TIGER/Line Shapefiles) with ArcGIS software.

### Cluster comparison

After completing the cluster analysis, we look at cluster differences for each of the clustering variables as well as differences in obesity and diabetes rates. To do this, we perform an Analysis of Variance (ANOVA) on the means of each cluster group for each variable to determine if an overall difference exists. If there is a difference, as evidenced by a small p-value, we then compare pairs of cluster means using a Post-Hoc Tukey Test. Both of these procedures are completed in R.

## Results

### Variable selection

Variable selection on the five standardized variables: unemployment, population density, median household income, socioeconomic status (SES) and low access to food determined that unemployment was not useful for clustering.

### Cluster results

**Fig. 3:**
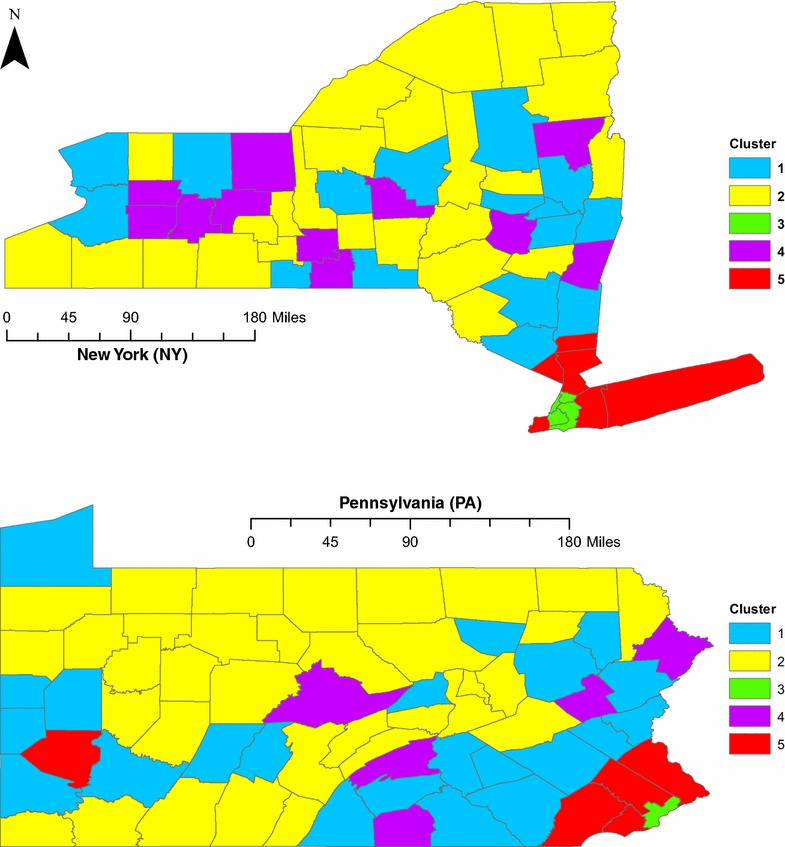
Clustering results for PA and NY. Five clusters were identified in NY (*top*) and PA (*bottom*). Although the clusters have heterogeneous spatial distributions, they can be classified according to the NCHS urban-rural classification codes of their component counties: clusters 1 (*blue*) and 4 (*purple*) comprise mostly small and non-metropolitan counties; cluster 2 (*yellow*) contains predominantly non-metropolitan counties; cluster 3 (*green*) is entirely large central metropolitan counties; and cluster 5 (*red*) includes large and large fringe metropolitan counties

Model-based clustering on the remaining four variables resulted in a clustering solution with the smallest BIC containing five clusters. The clustering solution for PA and NY is mapped in Fig. 3, and cluster sizes are given in Table 5. There are clear differences in the NCHS Urban-Rural Classification Scheme (see Table 3) for the different clusters. Cluster 1 (blue) is comprised of counties of all sizes, both non-metropolitan and metropolitan, however, this cluster predominately contains small and medium metropolitan counties. Cluster 2 (yellow) contains primarily non-metropolitan counties. Cluster 3 (green) is entirely large central metropolitan counties. Cluster 4 (purple) is similar to cluster 1 (contains large fringe to small metropolitan counties). Finally, the counties in cluster 5 (red) are large and large fringe metropolitan counties. This delineation of the clusters into metropolitan and non-metropolitan groups will be used throughout the results section and is summarized in Table 5.

**Table 5:** County count, NCHS classification and variable summaries for each cluster

	Cluster Number				
	1	2	3	4	5
No. of counties	38	59	5	16	11
NCHS class.	Small-medium metro.	Non-metro.	Large central metro.	Small–large fringe metro.	Large central and large fringe metro.
Population density	Low	Low	High	Low	Low
Median income	Medium	Low	Low	Medium	High
SES index	Low	Low	High	Low	Low
Low access	High	Medium	Low	Medium	High
Obesity rate	High	High	Low	High	Low
Diabetes rate	High	High	Medium	Medium	Low

Clustering variables described by whether on average the distribution is low, medium or high as compared to the other clusters

**Fig. 4:**
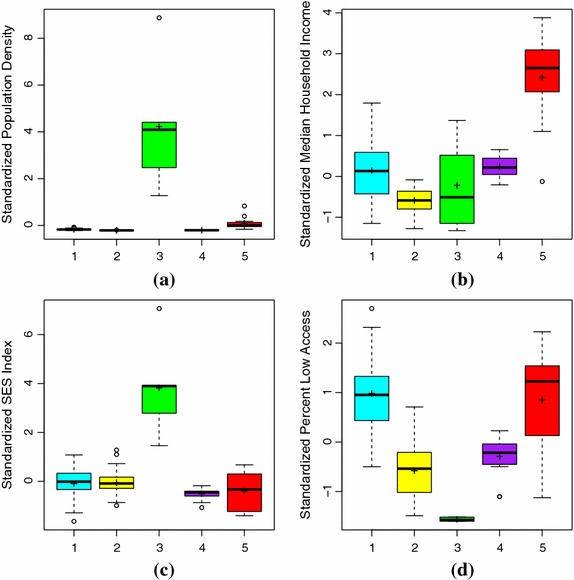
Comparison of standardized clustering variables across clusters for PA and NY. The variable selection process chose four variables to include in the clustering analysis: population density (**a**), median household income (**b**) deprivation as measured by our SES index (**c**) and percent county residents with low access to grocery stores or supermarkets (**d**), The distributions of these variables are shown via side-by-side *boxplots* disaggregated across the clusters, which are colored accordingly and labeled beneath each individual *boxplot*. Additionally, the mean for each cluster/variable combination is shown with a *plus sign*

We begin by first focusing on differences in clustering variables by cluster. There are four side-by-side boxplots for the clustering variables: population density (Fig. 4a), median household income (Fig. 4b), SES index (Fig. 4c) and low access to supermarkets (Fig. 4d), where the boxes are colored and labeled by cluster. Each individual boxplot extends from the minimum to maximum values that are not outliers, where any outliers are represented by open circles. The box contains the middle 50 % of the data, the median is the bold horizontal line and the mean is the plus sign. Table 5 provides the clustering variables with a description as to whether on average the distribution for that cluster is low, medium or high as compared to the other clusters for that variable.

There are significant differences across clusters for all four clustering variables (ANOVA p-value <0.001). The pairs of significant differences can be seen in Table 6. The cluster containing only large central metropolitan counties (cluster 3) has the lowest percentage of low food access—that is, a high proportion of the population has good access to supermarkets and grocery stores. This cluster’s level of food access is significantly different from all other clusters. Somewhat surprisingly, the next lowest percentage of low food access is in the non-metropolitan cluster (cluster 2). Cluster 5, containing large and large fringe counties had one of the highest percentages of low food access while also having a significantly higher median household income than that of the other four clusters. Further significant differences in income are between cluster 2 and clusters 1 and 4. Cluster 3 has the widest range of median household income values. This cluster, on average, is comparable to clusters 1 through 4. Finally, cluster 3 contains counties with significantly higher population densities and SES index scores.

**Table 6:** Pairs of significantly different clusters by variable for PA and NY from Tukey comparison (p-value <0.001)

Variable	Significant cluster pairs			
Population density	(1, 3)	(2, 3)	(3, 4)	(3, 5)
Median income	(1, 2)	(1, 5)	(2, 4)	(2, 5)
	(3, 5)	(4, 5)		
SES index	(1, 3)	(2, 3)	(3, 4)	(3, 5)
Low access	(1, 2)	(1, 3)	(1, 4)	(2, 3)
	(2, 5)	(3, 4)	(3, 5)	(4, 5)

### Predicting obesity and diabetes rates

**Fig. 5:**
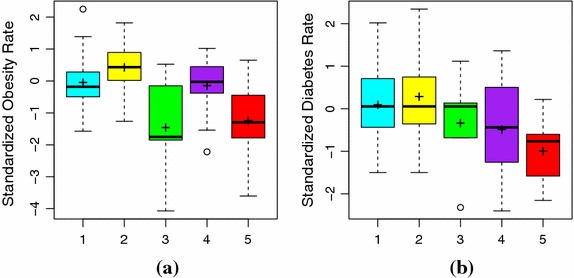
Comparing standardized obesity and diabetes rates by clusters for PA and NY. We examine the clusters’ predictive value for identifying counties with significantly higher rates of obesity (**a**) and diabetes (**b**). Clusters 3 (*green*) and 5 (*red*) are comprised of counties with lower obesity rates than those seen in the remaining clusters. While differences in diabetes prevalence are less pronounced, cluster 5 again stands out as exhibiting lower diabetes rates than those observed in other clusters

Recall that we are trying to determine if clustering the socioeconomic, demographic, and environmental characteristics of US counties in PA and NY allows us to distinguish differences in obesity and diabetes rates between counties. Fig. 5a shows side-by-side boxplots of the standardized obesity rate of each cluster while the standardized diabetes rates can be seen in Fig. 5b. Both clustering solutions produced clusters with an overall significant difference in obesity and diabetes rates (ANOVA p-value <0.001). The pairs of significant differences are given in Table 7. Clusters 3 and 5, containing only large central and large fringe counties have comparable obesity rates, that are significantly lower than the other 3 clusters. There are fewer significant differences observed in diabetes rates. We see that the clusters containing smaller metropolitan to non-metropolitan counties (clusters 1 and 2) have significantly higher diabetes rates than cluster 5 (large and large fringe). Additionally, cluster 2 has a higher diabetes rate than cluster 4. It is interesting to find that the large central metropolitan cluster (cluster 3) while having a significantly lower obesity rate from clusters 1 and 2, does not have a significantly lower diabetes rate.

**Table 7:** Pairs of significantly different clusters for PA and NY diet-related chronic disease rates from Tukey comparison (p-value <0.001)

Variable	Significant cluster pairs			
Obesity	(1, 3)	(1, 5)	(2, 3)	(2, 5)
	(3, 4)			
Diabetes	(1, 5)	(2, 4)	(2, 5)	

### Other variable selection procedures

As discussed in the “Methods” Section, we chose to use the variable selection procedure of Raftery and Dean because it resulted in a reduction of the variable space and produced practically and statistically meaningful clusters. The two variable selection procedures of Maugis et al. and Andrews and McNicholas kept all five variables and resulted in solutions containing 3 and 4 clusters respectively.

There was relatively high agreement between all three clustering solutions as the 3 and 4 cluster solutions resulted from merging clusters from our presented solution. The 3 cluster solution of Maugis et al. merges clusters 2 and 4 as well as clusters 3 and 5. Similarly, the 4 cluster solution of Andrews and McNicholas combines clusters 2 and 4 together while leaving clusters 3 and 5 separate. Since there is a significant difference between clusters 2 and 4 for the clustering variable median income and moreover for diabetes rate, we feel that it is important to keep these clusters separate. While there are not differences in obesity and diabetes rates for clusters 3 and 5, they differ significantly from each other in all four clustering variables. As mentioned previously and further commented on in the “Discussion”, this result is especially interesting for the low access to food variable. As such, we felt it necessary to keep cluster 3 and 5 separate and present the 5 cluster solution obtained using the variable selection procedure of Raftery and Dean.

## Discussion

This study applies model-based clustering to analyze county-level patterns in diet-related chronic disease. We are able to use socioeconomic status, household income, population density and access to food stores to identify two clusters—one of large central metropolitan counties and one large central and large fringe metropolitan counties–in both PA and NY with lower rates of obesity than counties with less dense populations.

Across both states, we see consistent evidence of clusters of large fringe and large central metropolitan counties with significantly lower rates of obesity than those seen in more rural or suburban clusters. This result is weaker for the examination of diabetes rates across clusters, although analysis of the separate states (see Additional file 1) suggests that this result may be affected by aggregation. Our result is in keeping with previous research showing a lower prevalence of obesity in urban counties [9]. In one of the low-obesity clusters, more people lack access to supermarkets and, in the other cluster, SES index scores are higher and median income is lower than one might expect, offering support for more complex relations between these variables and diet-related chronic disease than previously thought [44].

We also identify noteworthy patterns in the variables that distinguish each cluster. In particular, we find two clusters in both PA and NY that have a high proportion of residents who lack supermarket access. These clusters are generally comprised of medium or large fringe metropolitan areas, and the identified counties are also often adjacent to counties with higher levels of urbanization (as measured by the NCHS Urban-Rural Classification Scheme), which suggests that a significant portion of these clusters could be considered suburban. Research on disparities in access has largely focused on urban inner-city “food deserts” or gaps in rural areas with low car access [17], and some researchers have even argued that a “suburbanization” of grocery stores could be causing an increased prevalence of grocery stores in suburban areas [41]. Our contrasting result may be a product of the variable’s definition, which relies on a rural/urban dichotomy (see “Methods”). However, a similar clustering analysis conducted in Montreal, Quebec, also uncovered suburban clusters with very low levels of supermarket access [40]. Given these two results, further work may be necessary to understand the state of food access in suburban areas; policymakers would be well advised to be aware of potential food access problems in such counties.

This study has noteworthy strengths: the use of variable selection adds statistical rigor to the choice of variables; the model-based clustering analysis allows us both to identify subpopulations particularly at risk for diet-related chronic disease and to deconstruct the socioeconomic, demographic, and environmental characteristics that define these subpopulations; and our application of the Post-hoc Tukey Test allows us to identify the differences in these variables’ distributions. The method used in this paper is demonstrated in two states in part to ensure interpretability of the results, but the approach could be applied to large geographic areas. In addition, this method could be applied in other countries with comparable levels of obesity and diabetes to further explore factors contributing to the global rise of diet-related chronic disease.

The study is also subject to several limitations. Variables are assessed at the county level, which requires the aggregation of a number of variables collected at smaller scales and thus subjects the results to the effects of the modifiable areal unit problem [63]. This may lead to a scale effect that obscures variability in our SES index [64]. In addition, a finer grained analysis would have more power to detect distinct patterns in rural geographies. Finally, clustering results were presented for the combined counties of both states. We tested the robustness of the analysis on a disaggregated geographic area; while the major results (included in Additional file 1) are found to be robust once variables from PA and NY are separated, analysis done on a smaller geographic area does lead to the identification of fewer clusters.

Nevertheless, this study offers researchers an empirical means of identifying and classifying risk factors of value in predicting geographic patterns in diet-related chronic disease. We present a novel method for identifying the contributions of heterogeneously distributed risk factors to aggregate disease prevalence. Our application to obesity and diabetes provides evidence of the method’s predictive potential in associating socioeconomic, demographic, and environmental characteristics and population-level diet-related chronic disease prevalence.

## Conclusions

This study offers a generalizable and replicable method for the application of model-based clustering to the study of geographic patterns in rates of obesity and diabetes. We find two clusters of metropolitan counties with significantly lower rates of diet-related chronic disease than those seen in other county clusters, and we identify patterns of food access that are aligned with previous empirical work on the distribution of supermarkets in suburban areas. Our results demonstrate the utility of model-based clustering for the study of geographic disparities in obesity, diabetes, and other diet-related chronic diseases.

## Additional file

Additional file 1. Clustering results for the 129 counties disaggregated by state. Fewer clusters are found, however the overall cluster descriptions, as characterized by the NCHS codes and clustering variables are consistent with the aggregate clusters results presented.

## Abbreviations

PA: Pennsylvania
NY: New York
CDC: Centers for Disease Control and Prevention
BIC: Bayesian Information Criterion
BRFSS: Behavioral Risk Factor Surveillance System
NCHS: National Center for Health Statistics
SES: socioeconomic status
UPA: underprivileged area
